# Orthogonal Chemistry Enables Precision Nanoparticle Cofunctionalization for Tuning Immune Stimulation and Antigen Presentation

**DOI:** 10.1021/acs.biomac.5c02689

**Published:** 2026-06-03

**Authors:** Alexander J. Heiler, Claire A. McClain, Samuel N. Lucas, Guan Zhen He, M. G. Finn, Susan N. Thomas

**Affiliations:** † Parker H. Petit Institute for Bioengineering and Bioscience, 1372 Georgia Institute of Technology, Atlanta, Georgia 30332, United States; ‡ School of Chemical and Biomolecular Engineering, Georgia Institute of Technology, Atlanta, Georgia 30332, United States; § Wallace H. Coulter Department of Biomedical Engineering, Georgia Institute of Technology and Emory University, Atlanta, Georgia 30332, United States; ∥ School of Chemistry and Biochemistry, Georgia Institute of Technology, Atlanta, Georgia 30332, United States; ⊥ School of Biological Sciences, Georgia Institute of Technology, Atlanta, Georgia 30332, United States; # George W. Woodruff School of Mechanical Engineering, Georgia Institute of Technology, Atlanta, Georgia 30332, United States; ¶ Winship Cancer Institute, Emory University, Atlanta, Georgia 30322, United States

## Abstract

Combination therapies are increasingly utilized to treat complex diseases, capitalizing on drug synergies to potentiate overall therapeutic responses and improve patient outcomes. Drug delivery systems improve combination therapy access to tissues and cells of interest, but attempting to coconjugate multiple drugs to the same carrier can limit the precision of drug ratios or release behaviors, making it challenging to optimize the delivery of the individual drug compounds. Here, thiol–disulfide exchange and strain-promoted click chemistry are leveraged in combination with an established polymeric nanoparticle platform to achieve chemistry-defined control over both the conjugation ratio and release behavior of coconjugated moieties. When applied as a subunit vaccine platform, this system enables the modulation of lymph node dendritic cell maturation and antigen presentation. The results presented here thus demonstrate a versatile dual-functional drug delivery platform to overcome existing challenges in combination therapy delivery.

## Graphical abstract

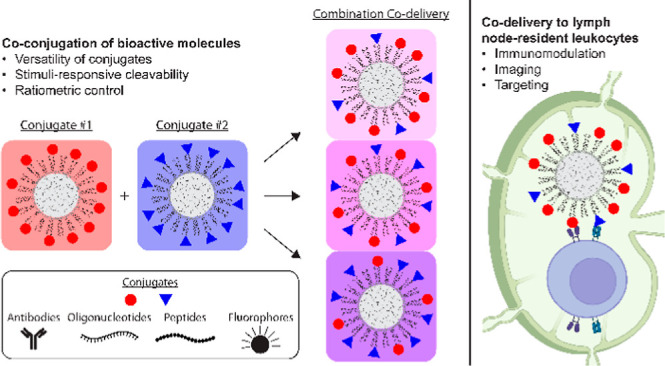

## Introduction

The treatment of complex and chronic diseases with combination therapies to surmount insufficient responses to individual therapies has increased in prevalence.^1–3^ Many diseases, including cancer,^4,5^ multidrug-resistant tuberculosis,^6^ hepatitis C,^7^ HIV,^8^ and hypertension,^9^ are now recognized to require multifaceted approaches to achieve durable results. The effectiveness of combination therapies can be further enhanced by codelivery of the pharmacological agents involved, eliciting synergistic activities^10^ or overcoming resistance mechanisms^11^ to maximize overall therapeutic efficacy. Drug codelivery technologies therefore offer the opportunity to improve disease outcomes in a multitude of therapeutic scenarios.

Many biomaterial systems and bioconjugation methods have been developed to facilitate codelivery while improving cargo bioavailability, decreasing systemic toxicity, increasing stability, and controlling therapeutic delivery through the formation of either permanent or stimuli-responsive, cleavable bonds.^12,13^ Despite these advantages, incorporating multiple therapeutic agents into one drug delivery system (DDS) can limit individual control over the delivery of each drug, an important concern as different drug components of a combination therapy may require unique optimal doses and release kinetics based on their modes of action.^14^ Some codelivery platforms utilize one reactive group to attach both conjugates, precluding the possibility of achieving independent stimuli-responsive retention and release. Additionally, in such platforms, the combination therapy components typically either maintain a constant relative dose^15–17^ or the relative dose is changed by mixing the components at different concentrations.^18,19^ Even systems that incorporate two conjugation chemistries often maintain a constant ratio of the conjugates.^20,21^ Therefore, there is a need for a platform that enables facile, ratiometrically controlled conjugation of two different molecular species while achieving independent tunability of release kinetics to improve combination delivery approaches. Such a system would have broad applicability across multiple therapeutic areas, including cancer and infectious disease.

One example wherein these design considerations are especially important is in the development of subunit nanovaccines, which are nanosized drug carriers that codeliver an antigen alongside a stimulatory adjuvant to activate antigen-presenting cells (APCs) to induce an antigen-specific immune response.^22^ As one example, Meng et al. demonstrated the modularity and generalizability of a nanovaccine platform, using different peptide antigens to elicit antigen-specific T-cell responses in multiple tumor models.^23^ However, to facilitate antigen presentation on APCs, the delivered antigen must be processed and loaded onto major histocompatibility complexes (MHCs), requiring separation from the delivery vehicle for maximal response.^24^ Additionally, the localized dosing of antigen and adjuvant components can modulate the degree of APC activation and antigen presentation,^25,26^ necessitating the design of codelivery systems in which both the conjugation extent and release behavior of the subunit vaccine components can be separately tuned to optimize antigen-specific immune responses.

Here, we employ poly­(propylene sulfide) nanoparticles (PPS-NPs), an established lymphatic system-targeting DDS that has been used to deliver cargo to immune cells within the lymph node,^27,28^ and introduce reactive groups onto the NP surface during synthesis through the use of functionalized Pluronic F127 copolymers that comprise the NP exterior. Previously, the pyridyl disulfide (PDS) functional group enabled facile and rapid conjugation of thiol-containing compounds through thiol–disulfide exchange (TDE),^29^ where the newly formed disulfide bond is susceptible to cleavage through reduction. These PDS-NP have been used in a variety of studies to examine immune cell responses to NP-conjugated small molecules,^27^ oligonucleotides,^30^ peptides,^31^ and antibodies.^32^ To achieve tunable coconjugation of multiple distinct compounds on a single NP, a second coupling chemistry was incorporated into the PPS-NP platform: strain-promoted azide–alkyne cycloaddition (SPAAC), where azido-NPs (N_3_-NP), synthesized using azido-Pluronic (N_3_-F127), readily conjugate dibenzocyclooctyne (DBCO)-containing molecules. While many different coupling chemistries are amenable for DDS conjugation,^33^ the modularity, high efficiency, mild reaction conditions, and formation of stable linkages of SPAAC make it ideal for our purpose.^34^ Most importantly, TDE and SPAAC are orthogonal: the thiol-PDS and azide-DBCO components do not cross-react or otherwise interfere with each other.

Both chemistries were simultaneously harnessed to increase the conjugation versatility of the PPS-NP platform. The resulting dual-functional NPs stably displayed a wide range of both homogeneous and heterogeneous compounds, including small molecules and IgG antibodies. This multiple conjugation strategy was controllable with regard to both drug release and ratiometric control. The dual-functional NPs were then leveraged as a model subunit vaccine, codelivering varying ratios of peptide antigen and oligonucleotide adjuvant to lymph node-resident APCs in order to modulate the extent of APC activation and antigen presentation. These results demonstrate the benefits of the dual-functional NPs for finely tuning drug delivery and response via exerting simultaneous control over ratiometric conjugation and release kinetics of multiple drug components.

## Experimental Section

All reagents and solvents were obtained from Sigma-Aldrich unless otherwise indicated. Pluronic mesylate, Pluronic thioether propionic acid methyl ester, and Pluronic thioether propionic acid sodium salt (COOH-F127) were all synthesized according to van der Vlies et al.^29^

### Synthesis of Pluronic Mesylate

Under argon, a solution of Pluronic F127 (23.6 g, 1.87 mmol) in anhydrous toluene (350 mL) was treated sequentially with triethylamine (2.6 mL, 18.6 mmol) and methanesulfonyl chloride (1.4 mL, 18.1 mmol), and the mixture was stirred for 18 h. The reaction mixture was then filtered, concentrated under reduced pressure, dissolved in dichloromethane (40 mL), and added dropwise to chilled diethyl ether (1000 mL) to induce precipitation of the desired product. The filtered white powder was washed with chilled diethyl ether and dried overnight.

### Synthesis of Pluronic Thioether Propionic Acid Methyl Ester

The Pluronic mesylate was dissolved in anhydrous dimethylformamide (100 mL) before addition of potassium carbonate (2.29 g, 16.6 mmol) and mercaptopropionic acid methyl ester (2 mL, 18.1 mmol). After stirring at room temperature for 20 h, the reaction mixture was concentrated under reduced pressure, dissolved in dichloromethane (100 mL) with activated carbon (11 g), filtered (washing the solid with additional dichloromethane), and precipitated into chilled diethyl ether (1000 mL). The filtered white powder was washed with chilled diethyl ether and dried overnight.

### Synthesis of Pluronic Thioether Propionic Acid Sodium Salt (COOH-F127)

The Pluronic thioether propionic acid methyl ester was dissolved in deionized water (200 mL) and stirred with a solution of sodium hydroxide (0.57 g) in deionized water (5 mL). The mixture was stirred for 21 h, and the desired product was isolated by dialysis in 3.5 kDa MWCO dialysis tubing (Spectrum Lab) against 3 × 5 L of deionized water over 1.5 days followed by lyophilization.

### Synthesis of Azido-Pluronic (N_3_-F127)

Azido-Pluronic was synthesized by the following protocol modified from that of Yan et al.^35^ Briefly, a solution of Pluronic mesylate in dimethylformamide (3.75 mL of solvent per 1 g of polymer) was treated with a solution of sodium azide (20 mol equiv with respect to polymer, 10 equiv to mesylate) in anhydrous dimethylformamide (1.25 mL of solvent per 1 g of polymer). The resulting mixture was heated at 65 °C for 16 h, cooled to room temperature, and filtered, and the insoluble material was washed with ethanol (10 mL). The combined filtrate was concentrated under reduced pressure and redissolved in deionized water (5 mL per 1 g of polymer). The aqueous solution was extracted with dichloromethane (3 × 10 mL), and the combined organic layers combined were dried with magnesium sulfate, filtered, and concentrated under reduced pressure to provide the desired azido-Pluronic.

### Poly­(propylene sulfide) Nanoparticle Synthesis

PPS-NPs were synthesized as previously described^29,36^ with slight modifications. Briefly, 5 wt % (250 mg) of COOH-F127, N_3_-F127, or a combination of the two was dissolved, with stirring at 1500 rpm, in degassed deionized water (5 mL) in a 25 mL two-neck round-bottom flask for 30 min. After degassing again, propylene sulfide (200 μL, 2.5 mmol, TCI Chemicals) was added under argon and stirred for 10 min. The initiator *S*,*S*′-(2,2-bis­((acetylthio)­methyl)­propane-1,3-diyl diethanethioate (7 mg, 0.02 mmol), activated by reaction with 161 μL of 25 wt % sodium methoxide in methanol for 15 min, was added to the round-bottom flask under argon and stirred for an additional 15 min. After addition of 1,8-diazabicyclo[5.4.0]­undec-7-ene (32 μL, 0.2 mmol) under argon, the reaction mixture was stirred for 24 h. After completion of the polymerization, the reaction mixture was exposed to air for 2 h to cross-link the NP core thiols. The NP solution was placed in 100 kDa MWCO cellulose membrane dialysis tubing (Spectrum Lab) and dialyzed against 4 × 5 L of deionized water for 2.5 days. The dialyzed NP solution was finally filtered through Acrodisc 0.2 μm filters (VWR). NP mass concentrations were determined by measuring the lyophilized mass from aliquots of known volume. A Zetasizer Nano ZS (Malvern Panalytical) was used to measure NP hydrodynamic size. NP solutions were diluted 50-fold in 1× PBS.

### PDS Functionalization of COOH-NP and COOH/N_3_-NP

The NP core thiols were rendered inert through reaction of 5 mL of NP with *N*-ethylmaleimide (NEM, 20 mg) in 1× PBS. The carboxylic acid groups on COOH-NP or COOH/N_3_-NP were functionalized with PDS as follows. 1-Ethyl-3-(3-(dimethylamino)­propyl)­carbodiimide (60 mg), *N*-hydroxysuccinimide (NHS, 60 mg), PDS cysteamine (Biosynth, 60 mg), and 2-(*N*-morpholino)­ethanesulfonic acid (108 mg, 100 mM) were added to NEM-capped NPs (5 mL) in 1× PBS and stirred for 18 h followed by dialysis against 4 × 5 L of deionized water over 2.5 days.

### Antibody Fluorophore Labeling

Alexa Fluor 647 NHS ester or Alexa Fluor 405 NHS ester (Thermo Fisher) was dissolved in DMSO (VWR) at 10 mg/mL. The fluorophore (6-fold molar excess) was added to the antibody in 1x PBS and rocked for 3 h. Fluorophore-labeled antibodies were purified from unreacted fluorophores using 3 successive 7 kDa MWCO Zeba Dye and Biotin Removal columns (Thermo Fisher), and the purification was validated using a Sepharose CL-6B size exclusion column (GE Healthcare).

### Antibody Functionalization

Thiol-functionalized antibodies were generated by reaction of antibodies with a 60-fold molar excess of Traut’s reagent (2-iminothiolane) in 1× PBS for 1 h and purification using a 7 kDa MWCO Zeba Desalting column (Thermo Fisher). Thiol incorporation was verified through increased absorbance at 412 nm after reaction with Ellman’s reagent, measured using a Synergy H4 BioTek Plate Reader. Dibenzocyclooctyne (DBCO)-functionalized antibodies were generated by reaction of antibodies with a 100-fold molar excess of DBCO-NHS ester linker (BroadPharm) in 1× PBS for 1 h. After purification using a 7 kDa MWCO Zeba Desalting column, DBCO-antibodies were further reacted with 20 molar excess of NEM for 3 h and purified using a Zeba Desalting column. DBCO incorporation was verified through increased absorbance at 309 nm measured using a Synergy H4 BioTek Plate Reader.

### NP Conjugation

Thiol-functionalized biomolecules (fluorophores, antibodies, or peptide), DBCO-functionalized biomolecules (fluorophores, antibodies, or CpG oligonucleotide), or a combination of both were mixed with functionalized NPs (PDS-NP, N_3_-NP, or PDS/N_3_-NP) and rocked overnight at room temperature. Conjugated NPs were separated from unreacted biomolecules through size exclusion chromatography (SEC) using Sepharose 4B (GE Healthcare) resin or sequential dilution and concentration (6 repetitions) using 30 kDa MWCO centrifugal filters (Amicon). SEC fractions were analyzed through various assays to detect the presence of NPs, antibodies, or fluorophores. Fractions containing conjugated NPs were pooled and concentrated using a 30 kDa MWCO centrifugal filter (Amicon). The thiol-functionalized Sun Fluor 650 was obtained from Nanocs, the DBCO-functionalized BP Fluor 405 was purchased from BroadPharm, the antibodies were obtained from Bio X Cell, the CSIINFEKL peptide was purchased from Biosynth, and the DBCO-functionalized CpG oligonucleotide (5′-TCC ATG ACG TTC CTG ACG TT-DBCO-3′) was purchased from Bio-Synthesis.

### Assays

A modified iodine assay^37^ was used to measure the NP concentration of conjugated NPs and detect NPs in SEC fractions. Briefly, samples were diluted 100-fold for conjugated NPs or 20-fold for SEC fractions. The diluted sample (200 μL) was mixed with a solution of barium chloride in 1N hydrochloric acid (50 μL, 50 mg/mL) and a 9:1 mixture of 20 mg/mL potassium iodide in water and 0.5 N I_2_ (25 μL). After reacting for 10 min, the absorbance was measured at 535 nm wavelength on a Synergy H4 BioTek Plate Reader. NP concentration was determined by comparison to a standard curve of NPs of known concentration. The Pierce Bicinchoninic Acid (BCA) assay (Thermo Fisher) was used to measure antibody concentrations. Briefly, samples were diluted 5-fold, and the diluted sample (25 μL) was mixed with BCA Buffer (200 μL), prepared according to kit guidelines. After incubation at 37 °C for 30 min, the absorbance was measured at 562 nm using a Synergy H4 BioTek Plate Reader. Antibody concentrations were determined by comparison to a standard curve of antibodies of known concentrations. Ellman’s assay was used to determine the concentrations of thiols. Briefly, an aliquot of the sample (20 μL) was mixed with a 4 mg/mL solution of Ellman’s Reagent in reaction buffer (20 μL, 0.1 M sodium phosphate and 1 mM EDTA in water, pH 8.0). After reacting for 15 min, the absorbance was measured at 412 nm using a Synergy H4 BioTek Plate Reader. Thiol concentrations were determined by comparison to a mercaptosuccinic acid standard curve. DBCO functionalization was verified through the direct measurement of DBCO groups. Samples were measured for their absorbance at 309 nm, and the concentration was determined by comparison to a standard curve of the DBCO-NHS ester linker. The functionalization of NPs with PDS was verified through the direct measurement of pyridine thione groups in the sample solution dissociated from the NP. The absorbance of pyridine thione, released from PDS/N_3_-NP after reaction with tris­(2-carboxyethyl)­phosphine (TCEP) (10 μL, 0.25 μmol), was measured at 340 nm using a Synergy H4 BioTek Plate Reader, and the concentration was determined by comparison to a pyridine thione standard curve. Fluorophores were measured directly to detect their presence in SEC fractions by their fluorescence or quantify their concentration on conjugated NPs by their absorbance, measured using the peak excitation and emission wavelengths for the respective fluorophore. Fluorophore concentrations were determined by comparison to a standard curve of the respective fluorophore. A modified fluorescamine assay was used to quantify the concentrations of peptides. Briefly, an aliquot of the sample (1 μL) was mixed with HEPES buffer (29 μL, 100 mM) and fluorescamine dissolved in acetone (4 μL, 5 mg/mL). After reacting for 10 min, the fluorescence with an excitation of 370 nm and emission of 490 nm was measured using a Synergy H4 BioTek Plate Reader, and concentrations were determined by comparison to a CSIINFEKL standard curve. A modified Gel Red assay was utilized to quantify oligonucleotide concentrations. An aliquot of the sample (5 μL) was diluted with 1× PBS (25 μL) and reacted with 2× Gel Red reagent (30 μL). After incubation for 10 min, the fluorescence was measured at an excitation of 300 nm and emission of 615 nm using a Synergy H4 BioTek Plate Reader. CpG concentrations were determined by comparison to a CpG standard curve of known concentration.

### Evaluating Conjugate Bond Permanence in Dual-Conjugated NPs

Dual-fluorophore NPs, dual-mAb NPs, or dual-CSIINFEKL/CpG-NPs were incubated with TCEP (25 mM) or a HEPES buffer control for 15 min. Three individual samples of dual-fluorophore NPs were each aliquoted into two solutions and treated with either TCEP or HEPES before the solutions were separated from released fluorophores using a 7 kDa MWCO Zeba Dye and Biotin Removal column (Thermo Fisher). The NP concentration was determined using the iodine assay, and the fluorophore concentrations were determined by measuring their respective absorbance at 400 and 650 nm using a Synergy H4 BioTek Plate Reader to calculate the number of fluorophores per NP. Dual-mAb NP samples were separated from released mAbs using a Sepharose 4B SEC column, where the eluted fractions were analyzed to detect NPs and mAbs. Dual-CSIINFEKL/CpG-NP samples were processed as the dual-fluorophore NPs but with CSIINFEKL concentration measured using a fluorescamine assay and CpG concentration measured using a Gel Red assay as described above.

### In Vivo Antigen and Adjuvant Codelivery

All animal procedures were approved by and followed guidelines set forth by the Institutional Animal Care and Use Committee of the Georgia Institute of Technology under animal protocol A100379. 30 μL of nanoparticles coconjugated with CSIINFEKL and CpG; mixtures of CSIINFEKL-conjugated nanoparticles and CpG-conjugated NPs; mixtures of unconjugated CSIINFEKL, CpG, and nanoparticles; or saline was administered intradermally to the forelimb, with *n* = 4–5 mice per group. Mice were euthanized, and the draining (ipsilateral) and nondraining (contralateral) lymph nodes were collected for analysis 24 h after injection.

### Flow Cytometry Analysis

Lymph nodes were incubated with 1 mg/mL of collagenase D at 37 °C for 1 h. Leukocytes were obtained by passing the lymph nodes through 70 μm cell strainers and washing with ice-cold 1× +/+ PBS. The entirety of the single cell suspensions was plated in 96 well U-bottom plates and blocked with Fc block (2.4G2) for 5 min on ice. Next, cells were stained with Zombie UV fixable viability dye at room temperature for 30 min and then antibody mixtures for surface staining on ice for 30 min. Cells were fixed using 2% paraformaldehyde on ice for 15 min, resuspended in FACS buffer, and analyzed using a Cytek Aurora flow cytometer and FlowJo. The antibodies used for flow cytometry staining are listed in Table S1.

## Results and Discussion

### Dual Conjugation to Dual-Functional Nanoparticles

Two orthogonal conjugation chemistries, TDE and SPAAC, were used to form dual-functional NPs. Pluronic F127 molecules bearing carboxylic acid and azide groups (COOH-F127 and N_3_-F127) were mixed in equal amounts during NP synthesis. The carboxy groups were subsequently functionalized with PDS groups to yield PDS/N_3_-NPs, ready for orthogonal bioconjugation (Figures 1A and S1 and S2). The resulting NPs retained the expected hydrodynamic diameter of 30–40 nm exhibited separately by the PDS-NP and N_3_-NP (Figure 1B), indicating stable integration of both functional groups onto the NP surface.

**Figure 1:**
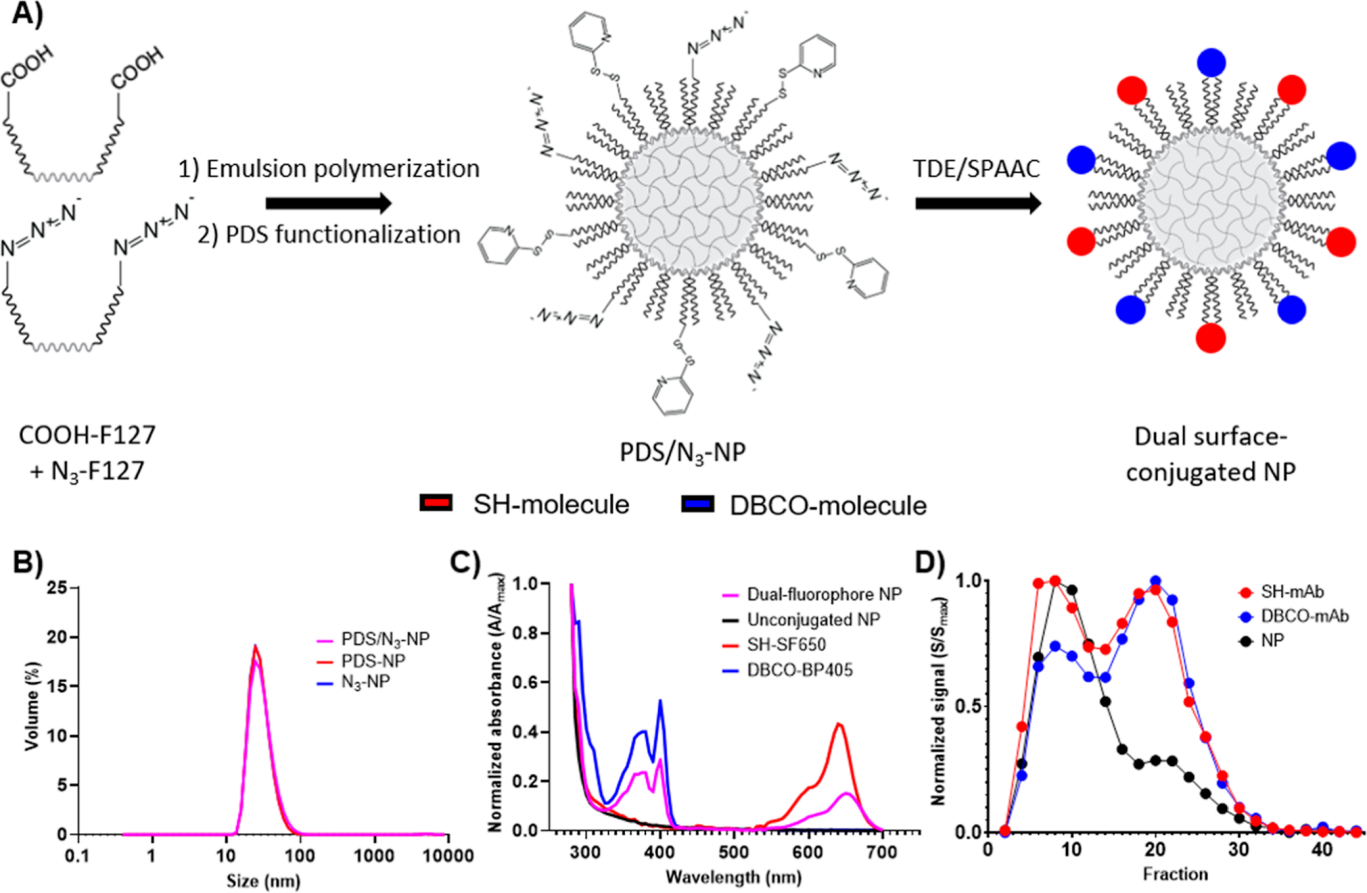
Dual-functionalized nanoparticles via orthogonal coupling chemistries. (A) Schematic of PPS-NP synthesis and functionalization of surface-stabilized Pluronics with two orthogonal functional groups and coconjugation using TDE and SPAAC. (B) Hydrodynamic diameter by volume of dual-functional PDS/N_3_-NP, PDS-NP, and N_3_-NP by dynamic light scattering. PDS/N_3_-NP was synthesized with equal parts COOH-F127 and N_3_-F127. (C) Absorbance spectrum of the purified dual-fluorophore NP, with the absorbance normalized by the maximum absorbance for each spectra. (D) Representative SEC elution of the dual-reactive NP and dual-mAb reaction mixture, with the absorbance (NP) and fluorescence (SH-mAb and DBCO-mAb) signals normalized by the maximum signal for each elution.

The TDE and SPAAC reactivities of the functionalized NPs were assessed with thiol- and -DBCO-bearing fluorophores as models of low-molecular-weight biomolecules and also representative imaging modalities. Thiol-functionalized Sun Fluor 650 (SH-SF650) and DBCO-functionalized BP Fluor 405 (DBCO-BP405) both successfully reacted with the PDS/N_3_-NP, as evidenced by the coelution of both fluorophores and NP at early fractions in SEC (Figure S3A). The conjugation chemistries progressed simultaneously without interference, demonstrating the one-pot reaction capability of this platform. The purified dual-conjugated product collected from the early fractions exhibited an absorbance spectrum with signatures of both fluorophores (Figure 1C), further illustrating successful dual conjugation.

Antibodies (mAbs), chosen as representative larger proteins with both therapeutic and targeting capacities, were similarly conjugated to the dual-functional NPs. IgG mAbs were labeled with Alexa Fluor 405 (AF405) and DBCO or Alex Fluor 647 (AF647) and thiols in order to distinguish the mAbs after conjugation (Figure S4A,B). After mixing the two labeled and functionalized mAbs with PDS/N_3_-NP, all three coeluted in SEC (Figure 1D), indicating successful dual-mAb conjugation to the NP. Since different vaccine components are often composed of different types of biomolecules, the heterogeneous conjugation of two different molecular entities was explored to evaluate the conjugation versatility of this system. Both small (fluorophores) and large (mAb) molecules could be simultaneously attached by the same procedure using both combinations (thiol-dye and DBCO-mAb; thiol-mAb; and DBCO-dye), highlighting the modularity of the system (Figure S3B,C). Thus, biologically relevant molecules spanning orders of magnitude of size were able to coconjugate via the orthogonal chemistries, demonstrating that the dual-functional NPs are a suitable platform for nanovaccine design.

### Coupling Chemistry-Dependent Stimuli Responsiveness of Conjugate Release from the Dual-Functionalized Nanoparticle

Combination therapy components may benefit from dual-stimulus-responsive and -unresponsive conjugation. In the case of vaccines, for example, antigens that need to be loaded onto MHC complexes for APC presentation must separate from the NP delivery vehicle, while TLR ligand adjuvants display increased avidity afforded by permanent conjugation on the NP surface. Therefore, the dual-functional NPs were evaluated for tunable cleavage facilitated by the orthogonal TDE and SPAAC chemistries (Figure 2A). First, dual-fluorophore NPs were examined for their susceptibility to reduction via tris­(2-carboxyethyl)­phosphine (TCEP), modeling the reductive environment cell-internalized NPs experience in the cell cytoplasm.^38^ After incubation with TCEP and cleaning using dye removal columns, there was reduced signal from the fluorophore conjugated via TDE, indicating cleavage of the disulfide bond and release of the conjugated fluorophore, while the SPAAC-conjugated fluorophore signal remained constant (Figure 2B) due to the physiologically stable triazole linkage,^39^ which is unresponsive to external stimuli. The fact that only approximately 25% of the disulfide-conjugated fluorophore was removed likely reflects incomplete accessibility of the disulfide bonds to TCEP due to steric hindrance or oxidation of TCEP in the physiologically mimicking phosphate-buffered solution. Nonetheless, the cleavability of the disulfide and permanence of the triazole demonstrate chemistry-specific release of the conjugates from the NP.

**Figure 2:**
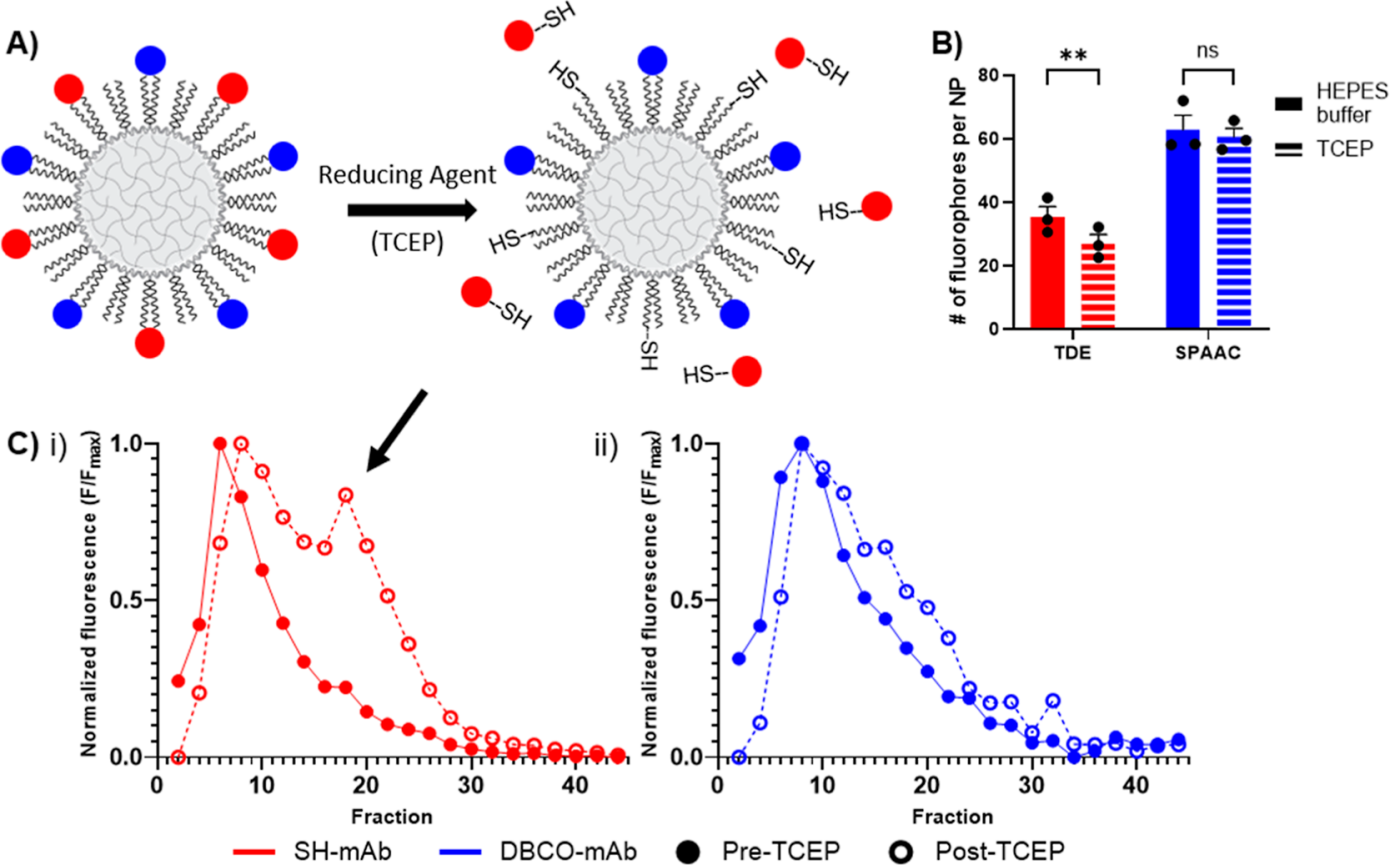
Orthogonal chemistries yield cleavable and irreversible linkages on the dual-conjugated nanoparticle. (A) Schematic of disulfide bond reduction and SPAAC bond retention in the dual-conjugated NP. (B) Retained fluorophores per NP with and without disulfide reduction. *n* = 3. ** (*p* < 0.01) indicates significant difference by multiple paired *t* tests with Holm–Šidák’s multiple comparison test. (C) SEC elution of dual-mAb NP, with and without disulfide reduction, comparing (i) SH-mAb and (ii) DBCO-mAb. Fluorescence was normalized by the maximum fluorescence for each elution, and the black arrow indicates the released SH-mAb. Precursor PDS/N_3_-NP was synthesized using equal parts COOH-F127 and N_3_-F127.

Similarly, the responsiveness of the coupling chemistries was evaluated for the dual-mAb NPs. Without incubation with a reducing agent, both mAbs eluted with the NP (around fraction 10 when evaluated using SEC, Figures 1D and 2C). However, when incubated with TCEP, the thiolated mAb was released, indicated by the appearance of an additional later peak (around fraction 18), while the DBCO-mAb remained attached to the NP under both reducing and nonreducing conditions. As with the dual-fluorophore NP experiment, some SH-mAb remained attached to the NP due to incomplete disulfide cleavage with TCEP. Overall, the combination of TDE and SPAAC incorporated into one NP grants control over the retention and release of bioactive molecules conjugated to the NP, enabling differential delivery dynamics for combination delivery systems, such as nanovaccines.

### Ratiometric Control over Biomolecule Conjugation

The modular nature of functional group incorporation using different Pluronic components provides a convenient way to provide ratiometric control, an ideal approach for separately modulating the relative dose of coconjugated biomolecules. COOH-F127 and N_3_-F127 were mixed during NP synthesis at ratios of 90/10, 50/50, 30/70, and 10/90 by mass, and the carboxylic acid groups on the resulting particles were converted to PDS groups as before. The sizes of the resulting functionalized PDS/N_3_-NPs were unaffected by the different blends of the functionalized copolymer (Figure 3A), and each NP exhibited the expected relative amounts of PDS and N_3_ groups (Figure 3B–D) on the NP surface, demonstrating that the dual-surface functionalization of the NPs can be finely controlled during NP synthesis without affecting the other bulk NP properties.

**Figure 3:**
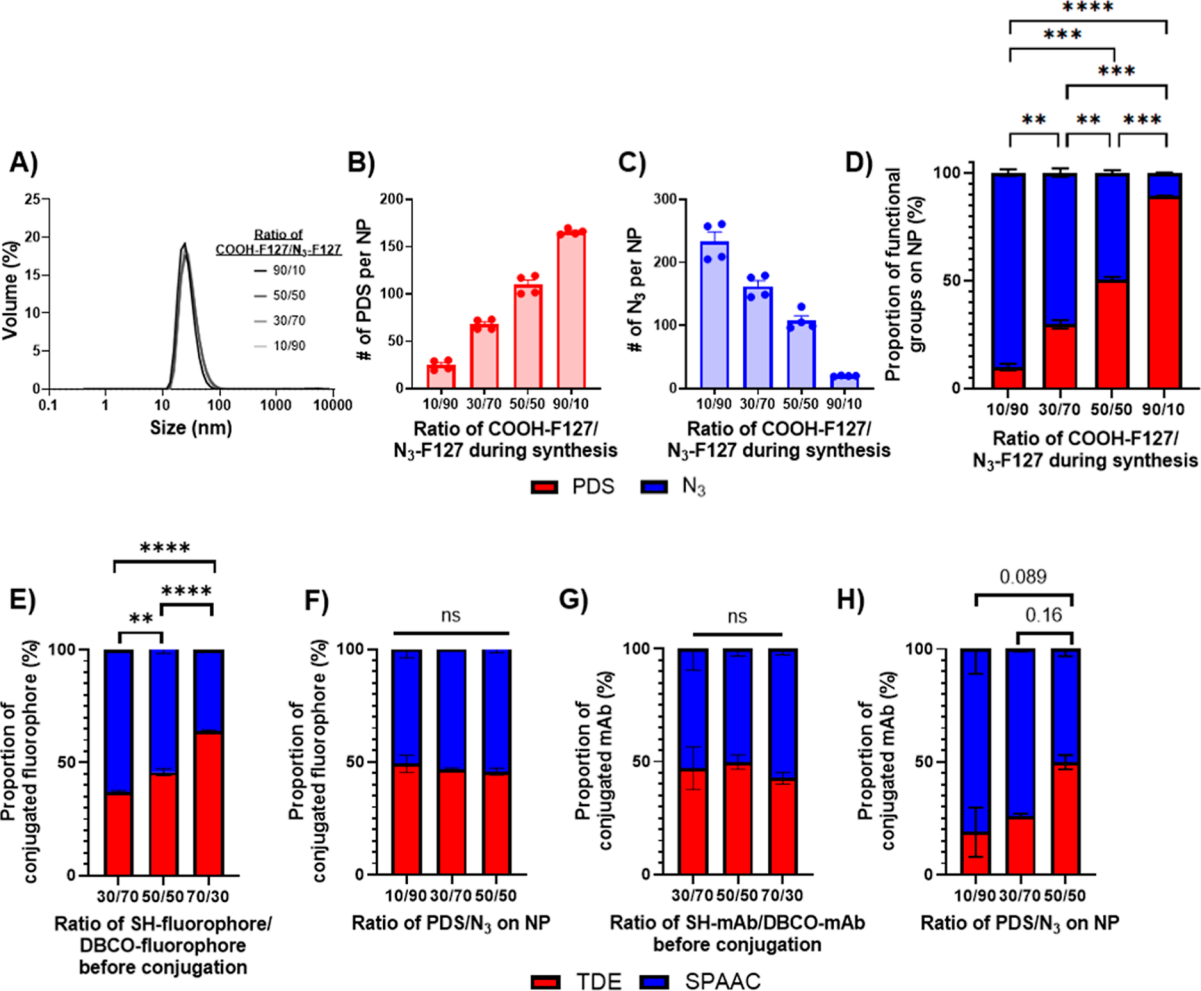
Ratio of dual conjugates is controlled by nanoparticle composition. (A) Hydrodynamic diameter by volume of dual-functional NPs synthesized using various blends of COOH-F127 and N_3_-F127 copolymers by dynamic light scattering. (B) Number of PDS groups per NP released by disulfide bond reduction. *n* = 4 technical replicates across 2 independent experiments. (C) Number of N_3_ groups per NP, measured by conjugation of AF647-DBCO. *n* = 4 technical replicates across 2 independent experiments. (D) NP surface functionalization dependence on copolymer reagent, determined as the percentage of each functional group comprising the total number functional groups on the NP surface. *n* = 4 technical replicates across 2 independent experiments. (E,F) Ratio of conjugated fluorophores for (E) 50/50 PDS/N_3_-NP conjugating different ratios of functionalized fluorophores and (F) NPs with different ratios of functional groups conjugating a 50/50 mixture of functionalized fluorophores. *n* = 3. (G,H) Ratio of conjugated mAbs for (G) 50/50 PDS/N_3_-NP conjugating different ratios of functionalized mAbs and (H) NPs with different ratios of functional groups conjugating a 50/50 mixture of functionalized mAbs. *n* = 2. * (*p* < 0.05), ** (*p* < 0.01), *** (*p* < 0.001), and **** (*p* < 0.0001) indicate significant difference by one-way ANOVA with Tukey’s multiple comparison test.

Having characterized the functionalization of the NPs with PDS and N_3_ groups and demonstrated precise control over the ratio of these groups, we next evaluated the ratio of conjugation. Small-molecule fluorophores were reacted in excess of the functional groups on the NP, either varying the ratio of the fluorophores while maintaining a constant ratio of the NP functional groups or keeping a constant fluorophore ratio while changing the NP functional group ratios. The ratio of fluorophores that conjugated to the NP depended on the ratio of the fluorophores reacted (Figure 3E) but did not change with different NP functional group ratios (Figure 3F). Altering the ratio of the reacting fluorophores changed the abundance of each fluorophore conjugated compared with the NP functional groups. Increasing the fluorophore excess increased the extent of conjugation until plateauing around 2-fold excess, where further saturating the NP functional groups did not lead to additional conjugation (Figure S5A). Therefore, either decreasing the excess of the SH-fluorophore at the 30/70 SH-fluorophore/DBCO-fluorophore ratio or decreasing the excess of the DBCO-fluorophore at the 70/30 ratio resulted in less conjugation of the fluorophore in question, altering the overall conjugation ratio (Figure S5B). With the different NP functional group ratios, increasing the number of N_3_ groups, in turn, decreasing the DBCO-fluorophore excess, slightly decreased the number of DBCO-fluorophores conjugated without significantly affecting the overall ratio of the conjugated fluorophores (Figure S5C). Additionally, decreasing the number of PDS groups, which increased the fluorophore excess, resulted in no additional fluorophore conjugation because the PDS groups were already saturated. Overall, for bioactive molecules conjugated at high extents, such as small molecules, the conjugation ratio is controlled through the saturation of the DDS reactive groups.

To examine ratiometric conjugation of larger molecules, two mAbs were coconjugated to the NPs, similarly varying either the reacting mAb ratio or the NP functional group ratio. Notably, the NP functional groups were in excess of the reacting groups displayed on the mAbs except when utilizing NPs with low extents of PDS functionalization. The mAb conjugation ratio depended on the NP functional group ratio rather than on the ratio of the reacting mAbs (Figure 3G,H) and was further correlated to the excess of the NP functional groups over the reactive groups on the mAbs (Figure S5D), indicating that mAb conjugation is controlled by the availability of the functional groups on the NP. When reacting different ratios of SH-mAb and DBCO-mAb, the total number of conjugated mAbs varied, but the extent of conjugation changed similarly for both SH-mAb and DBCO-mAb (Figure S5E). NPs with a constant ratio of functional groups had similar extents of functional group excess over the mAb reactive groups and overall conjugated to a constant ratio of mAbs (Figure 3G). With varied NP functional groups, as the number of PDS groups decreased and the number of N_3_ groups increased, the number of conjugating SH-mAbs remained constant, while the number of conjugating DBCO-mAbs increased (Figure S5F). A higher excess of the NP-displayed N_3_ groups to the DBCO groups on the mAb enabled more mAb conjugation, skewing the conjugation ratio toward DBCO-mAb (Figure 3H) and further illustrating that the conjugation of larger bioactive molecules is modulated by the prevalence of the functional groups on the DDS. Due to potential vaccine components ranging over orders of magnitude in size, from proteins to peptides, DNA, RNA, and lipopolysaccharides, using NP chemistry to modulate the extents of conjugation facilitates the separate regulation of the concentrations of codelivered vaccine subunits.

### Ratio of Nanoparticle-Mediated Antigen and Adjuvant Codelivery Tunes In Vivo Immune Response Potency

Having successfully demonstrated the tunable nature of the dual-functional NP system to coconjugating specified ratios of components along with controlled release, relevant moieties were combined in a nanovaccine model. We and others have demonstrated that peptides conjugated to nanoparticles via TDE are readily available for antigen presentation both *in vitro* and *in vivo*.^40,41^ Therefore, a thiol-containing derivative of the model ovalbumin MHC class I peptide antigen SIINFEKL was conjugated to the NP via TDE, enabling release in the reductive intracellular environment of APCs, while the model adjuvant CpG, a TLR9 ligand, was conjugated to the NP via DBCO-N_3_ click chemistry. PDS/N_3_-NPs with PDS/N_3_ ratios of 10/90, 50/50, and 90/10 were utilized to synthesize CSIINFEKL/CpG-NP formulations bearing low, medium, and high amounts of CSIINFEKL, respectively, while CpG conjugation was kept constant across formulations to control for its contribution within the overall immune response to coconjugated antigen and adjuvant (Figure 4B,C). To confirm reduction sensitivity of the CSIINFEKL antigen, CSIINFEKL and CpG conjugated to the NP were measured before and after TCEP reduction. All formulations exhibited reduction-triggered release of CSIINFEKL and stable conjugation of CpG, though only medium and high CSIINFEKL formulations saw significant reductions in CSIINFEKL conjugation (Figure S6). CSIINFEKL/CpG-NPs were then administered intradermally in the mouse forelimb to drain to the axillary and brachial lymph nodes (Figure 4A). Control formulations were also administered, composed of a mixture of single-conjugated CSIINFEKL-NP and CpG-NP or unconjugated CSIIFNEKL, CpG, and NPs, all of which were dose-matched to the dual-conjugated NPs with medium amounts of CSIIFNEKL conjugation.

**Figure 4:**
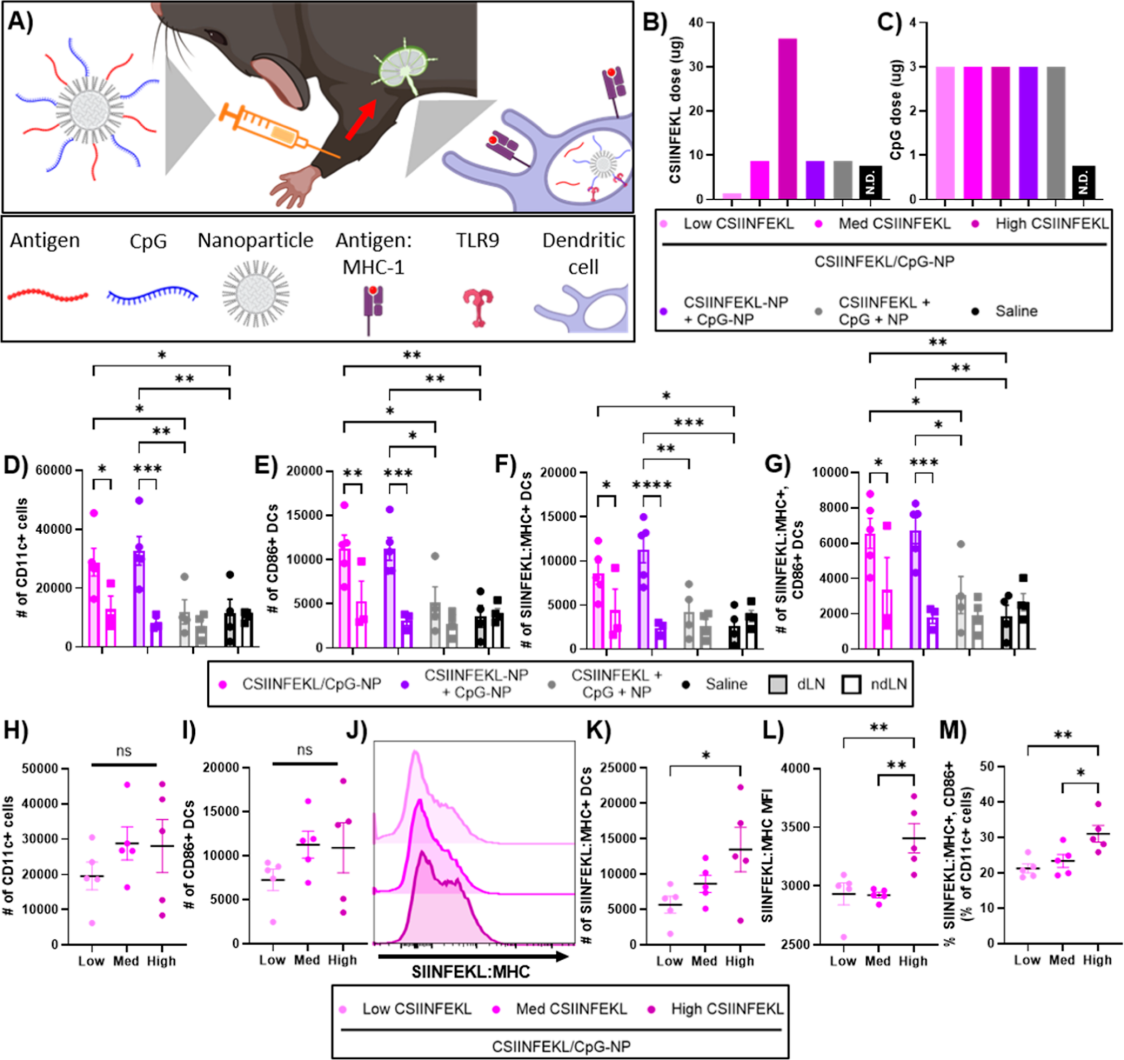
Ratio of nanoparticle codelivery of antigen and adjuvant modulates number of total and activated dendritic cells and their extent of antigen presentation. (A) Schematic of dual-antigen/adjuvant-NP delivery to lymph node-resident dendritic cells. (B,C) Amount of (B) CSIINFEKL and (C) CpG administered via the dual-conjugated, single-conjugated, or unconjugated NPs. (D–G) Number of (D) CD11c^+^ cells and (E) CD86^+^, (F) SIINFEKL:MHC^+^, and (G) SIINFEKL:MHC^+^, CD86^+^ DCs in the dLN and ndLN of mice treated with dose-matched dual-conjugated, single-conjugated, and unconjugated NPs. (H,I) Number of (H) CD11c^+^ cells and (I) CD86^+^ DCs in the dLN of mice treated with dual-conjugated NPs with varying CSIINFEKL conjugation. (J) Representative flow cytometry histograms of SIINFEKL:MHC on DCs in the dLN, normalized to the mode. (K–M) (K) Number of SIINFEKL:MHC^+^ DCs, (L) SIINFEKL:MHC median fluorescence intensity on DCs, and (M) frequency of SIINFEKL:MHC^+^, CD86^+^ DCs in the dLN of mice treated with dual-conjugated NPs with varying CSIINFEKL conjugation. (D–G) * (*p* < 0.05), ** (*p* < 0.01), *** (*p* < 0.001), and **** (*p* < 0.0001) indicate significant difference by two-way ANOVA with Tukey’s multiple comparison test, with *n* = 3–5. (H, I, and K–M) * (*p* < 0.05), ** (*p* < 0.01), *** (*p* < 0.001), and **** (*p* < 0.0001) indicate significant difference by one-way ANOVA with Tukey’s multiple comparison test, with *n* = 5.

24 h after NP injection, the DC response was analyzed in the draining lymph nodes (dLN) and nondraining lymph nodes (ndLN) (Figure S7). There were significant enhancements in the number, type, and antigen presentation of DCs in the dLN, but not in the ndLN, when the antigen and adjuvant were carried by the same or different NPs, as shown in Figure 4. Thus, of the larger number of DCs in the dLN (Figure 4D), a greater number expressed CD86 and SIINFEKL-bound MHC-I in response to the dual-conjugated or mixed single-conjugated NPs compared to unconjugated or saline groups (Figure 4E,F). The significant number of SIINFEKL:MHC^+^ DCs demonstrated that the TDE-conjugated antigen was readily available for presentation, despite the partial disulfide cleavage seen *in vitro*. Furthermore, the dLN also contained more DCs coexpressing CD86 and SIINFEKL-bound MHC-I (Figure 4G), providing the necessary cues to activate naïve T cells. Therefore, this nanovaccine platform demonstrates efficacy in increasing not only DC populations with enhanced maturation markers but also peptide:MHC loading on DCs in the dLNs consistent with locoregional controlled delivery.

While DCs were costimulated by and presented antigen from either the dual-conjugated or mixed single-conjugated NPs, the dual-conjugated NPs enabled control over the localized doses of the codelivered CSIINFEKL and CpG. As the administered CpG dose was constant across formulations, different CSIINFEKL/CpG-NP ratios gave rise to comparable numbers of total (Figure 4H) and activated DCs (Figure 4I) in the dLNs. However, increasing the extent of CSIINFEKL conjugation did provide greater numbers of DCs expressing SIINFEKL-bound MHC I (Figure 4J,K). The fact that different extents of CSIINFEKL conjugation gave rise to different numbers of SIINFEKL:MHC^+^ DCs but not different numbers of CD86^+^ DCs therefore suggests that DCs access the dual-conjugated NPs similarly between formulations, resulting in similar extents of DC access to CpG but different extents of DC access to SIINFEKL depending on the extent of CSIINFEKL conjugation. In addition, the DCs exposed to dual-conjugated NPs with high CSIINFEKL conjugation exhibited higher SIINFEKL-bound MHC-I median fluorescence intensity (MFI) and a higher frequency of DCs coexpressing CD86 and SIINFEKL-bound MHC-I (Figure 4L,M). These results demonstrate modulation of the degree of DC antigen presentation through control over the nanovaccine coconjugation. Overall, this DDS, capable of varying the relative dose of delivered antigen via the surface conjugation chemistry, can tune the potency of antigen-specific immune stimulation.

While passively targeting the dLNs, the dual-conjugated NPs are not targeted to a specific immune cell within the LN and can associate with other LN APCs, such as B cells. The total number of B cells and the number of SIINFEKL-antigen-presenting B cells were only elevated in mice treated with the mixed single-conjugated NPs (Figure 5A,C), suggesting that NP codelivery of antigen and adjuvant interacts differently with DCs than B cells and that B cells may require an alternate combination of signals due to their different distribution within the lymph node^42^ reduced cross presentation capacity relative to DCs.^43^ However, there were a greater number of CD86^+^ B cells and B cells coexpressing CD86 and SIINFEKL-bound MHC-I in the dLN in response to the dose-matched CSIINFEKL/CpG-NP and mixed CSIINFEKL-NP and CpG-NP, in comparison to saline (Figure 5A–D). Due to the similar extents of CpG delivered by the different dual-conjugated NPs, the total number of B cells and activated B cells were similar between formulations (Figure 5E,F). However, CSIINFEKL/CpG-NPs with high CSIINFEKL conjugation elicited greater frequencies of B cells expressing SIINFEKL-bound MHC-I, including B cells coexpressing SIINFEKL-MHC-I and CD86, while also increasing the extent of antigen presentation as measured by SIINFEKL-bound MHC-I MFI (Figure 5G–I). Overall, B cells experienced both activation and dose-dependent antigen presentation, demonstrating that the response to the dual-conjugated NPs extends beyond DCs to other types of APCs within the dLNs. Furthermore, the dual-conjugated CSIINFEKL/CpG-NPs promoted a relatively higher response in DCs, while the mixed CSIINFEKL-NP and CpG-NP exhibited a greater proportion of activated and antigen-presenting B cells (Figure 5J–L), indicating that the mechanism of vaccine component codelivery, either coconjugated or separately conjugated to NPs, alters the nanovaccine distribution within the lymph node toward DCs or B cells, respectively. DCs have been described as the primary APCs involved in cross-presentation to CD8^+^ T cells,^44^ suggesting that the coconjugation of antigen and adjuvant onto one NP may induce a more robust immune response in comparison to coadministered but separately conjugated nanovaccine components.

**Figure 5:**
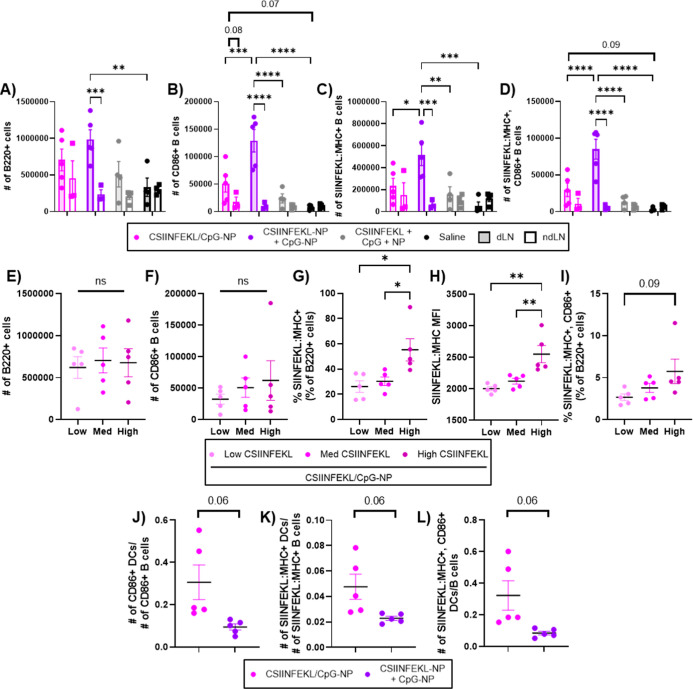
Ratio of nanoparticle codelivery of antigen and adjuvant tunes delivery to B cells. (A–D) Number of (A) B220^+^ cells, (B) CD86^+^ B cells, (C) SIINFEKL:MHC^+^ B cells, and (D) SIINFEKL:MHC^+^, CD86^+^ B cells in the dLN and ndLN of mice treated with dual-conjugated, single-conjugated, and unconjugated NPs. (E–I) (E) Number of B220^+^ cells, (F) number of CD86^+^ B cells, (G) frequency of SIINFEKL:MHC^+^ B cells, (H) SIINFEKL:MHC MFI on B cells, and (I) frequency of SIINFEKL:MHC^+^, CD86^+^ B cells in the dLN for mice treated with dual-conjugated NPs with varying CSIINFEKL conjugation. (J–L) Ratio of the number of DCs to the number of B cells in the dLN expressing (J) CD86, (K) SIINFEKL:MHC, and (L) coexpressing CD86 and SIINFEKL:MHC. (A–D) * (*p* < 0.05), ** (*p* < 0.01), *** (*p* < 0.001), and **** (*p* < 0.0001) indicate significant difference by two-way ANOVA with Tukey’s multiple comparison test, with *n* = 3–5. (E–I) * (*p* < 0.05), ** (*p* < 0.01), *** (*p* < 0.001), and **** (*p* < 0.0001) indicate significant difference by one-way ANOVA with Tukey’s multiple comparison test, with *n* = 5. (J–L) *p* values measured by Welch’s *t*-test, with *n* = 5.

The ability to tune the extent of APC antigen presentation by tuning the relative coconjugation of peptide antigen to the NP surface has the potential to expand groups of T cells with phenotypes optimized for different diseases. As demonstrated by Ng et al., antigen doses delivered beyond an optimal level can attenuate the CD8^+^ T-cell response, highlighting the importance of controlling the delivered dose to elicit a robust immune response.^45^ Moreover, APC antigen density can modulate T-cell differentiation, with low antigen densities inducing effector CD8^+^ T cells for cytotoxicity and high antigen densities promoting differentiation to a mixture of effector and memory phenotypes for long-term immunity.^46^ As Kim et al. demonstrated, cytotoxic T cells with different sensitivities to recognize and lyse their target cells can be expanded in response to splenocyte antigen presentation induced from different antigen doses, with low antigen doses promoting cytotoxic T cells with high sensitivities.^47^ Similarly, the effector function of T cells has been demonstrated to depend on the degree of stimulation from APCs, balancing between understimulated inertness and overstimulated exhaustion, with an optimal amount of stimulation maximizing the T-cell response.^48^ Therefore, the dual-conjugated NP platform can potentially enable nanovaccine tuning to expand T cells with different phenotypes and sensitivities optimal for different diseases: for example, delivering high antigen doses to promote long-term protection against repeated exposure to viral pathogens or conjugating low extents of antigen to expand highly sensitive T cells capable of recognizing suboptimal targets,^49^ including cancer cells with limited or downregulated expression of peptide-bound MHC complexes in the immunosuppressive tumor microenvironment.^50^ Overall, the ability to not only tune the antigen dose but also tune it separately from other vaccine components, imparted by the orthogonal chemistries utilized to design the CSIINFEKL/CpG-NPs, may be advantageous in optimizing nanovaccine formulations to tune the quality of the elicited immune response.

## Conclusion

This study designed dual-functional NPs leveraging orthogonal conjugation chemistries for the simultaneous coattachment of biomolecules with heterogeneity in both their class and size. These NPs exhibited control over not only the stimuli-responsive retention or release of the conjugates but also the relative extent of conjugation of the attached bioactive molecules. The dual-functional PDS/N_3_-NP system was utilized to codeliver an oligonucleotide adjuvant and different doses of peptide antigen to modulate the degree of antigen presentation by lymph node-resident APCs, exemplifying how biomaterial approaches to vaccine delivery provide opportunity to modulate the APC response and, in turn, potentially the downstream T-cell-mediated response. Beyond nanovaccines, the capability of separately tuning the localized delivery of coconjugated compounds to nanoparticles can be utilized to enhance currently used combination biomolecule-based therapeutic and diagnostic applications, such as coadministered immune checkpoint inhibitors and chemotherapy that improve response rates in cancer patients,^51^ targeting antibodies coupled with cytotoxic drugs for targeted antitumor activity,^52^ or theranostics that combine therapeutics with imaging agents for simultaneous disease diagnosis and treatment.^53^ The enhanced synergy promoted by this engineered coconjugation DDS can facilitate not only subunit approaches to vaccination and but also more general combination therapies for multifaceted approaches to complex diseases.

## Supplementary Material



## Notes

SPAAC NP conjugation characterization, SEC analysis of dual conjugation, antibody functionalization, fluorophore and antibody extent of dual conjugation, responsiveness of dual CSIINFEKL/CpG-NP to TCEP reduction, and flow cytometry gating strategy (PDF)

## Abbreviations

DDS: drug delivery system
ICI: immune checkpoint inhibitor
ADC: antibody–drug conjugate
PPS-NP: poly­(propylene sulfide) nanoparticle
NP: nanoparticle
DC: dendritic cell
PDS: pyridyl disulfide
TDE: thiol–disulfide exchange
SPAAC: strain-promoted azido-alkyne cycloaddition
DBCO: dibenzocyclooctyne
N3-F127: azido-Pluronic
N3-NP: azido-nanoparticle
COOH-F127: Pluronic thioether propionic acid sodium salt
SH-SF650: Sun Fluor 650
DBCO-BP405: DBCO-functionalized BP Fluor 405
SEC: size exclusion chromatography
mAb: antibody
AF405: Alexa Fluor 405
AF647: Alexa Fluor 647
dLN: draining lymph node
ndLN: nondraining lymph node
MFI: median fluorescence intensity
APC: antigen-presenting cell
MHC: major histocompatibility complex
NEM: *N*-ethyl maleimide
NHS: *N*-hydroxysuccinimide
BCA: bicinchoninic acid
TCEP: tris­(2-carboxyethyl)­phosphine
DLS: dynamic light scattering
