# Supplementary material for: Orthogonal Chemistry Enables Precision Nanoparticle Cofunctionalization for Tuning Immune Stimulation and Antigen Presentation
Source: Biomacromolecules. 2026 Jun 3;27(7):4228–39. doi: 10.1021/acs.biomac.5c02689 (PMC13370783; doi:10.1021/acs.biomac.5c02689)
Supplement: Supplementary file 1 [file bm5c02689_si_001.pdf]

Supplementary Information

Orthogonal chemistry enables precision nanoparticle co-functionalization for tuning immune stimulation and antigen presentation

Alexander J. Heiler[†,‡], Claire A. McClain[†,§], Samuel N. Lucas[†,§], Guan Zhen He[†,§], M.G. Finn[#,Δ], and Susan N. Thomas[†,§,¶,]*

[†]Parker H. Petit Institute for Bioengineering and Bioscience, Georgia Institute of Technology, Atlanta, GA 30332, United States of America

[‡]School of Chemical and Biomolecular Engineering, Georgia Institute of Technology, Atlanta, GA 30332, United States of America

[§]Wallace H. Coulter Department of Biomedical Engineering, Georgia Institute of Technology and Emory University, Atlanta, GA 30332, United States of America

[#]School of Chemistry and Biochemistry, Georgia Institute of Technology, Atlanta, GA 30332, United States of America

[Δ]School of Biological Sciences, Georgia Institute of Technology, Atlanta, GA 30332, United States of America

George W. Woodruff School of Mechanical Engineering, Georgia Institute of Technology, Atlanta, GA 30332, United States of America

[¶]Winship Cancer Institute, Emory University, Atlanta, GA 30322, United States of America

*Corresponding Author

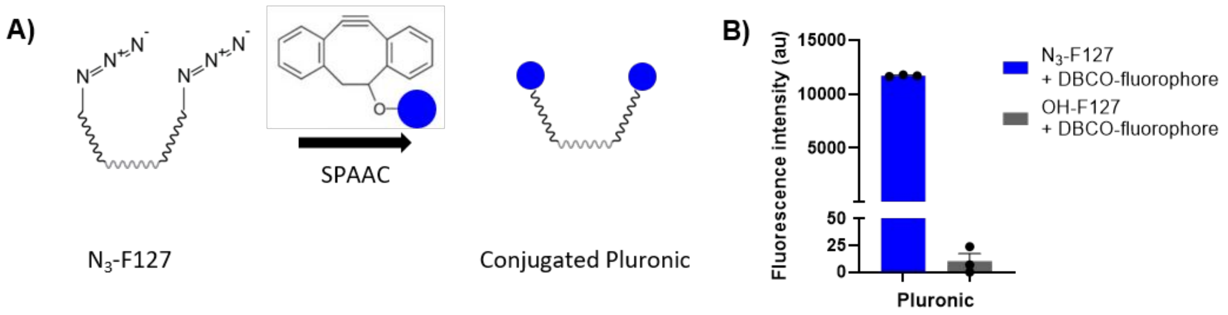

Figure S1. N_3-F127 reactivity. A) Schematic of N_3-F127 reaction with a DBCO-functionalized compound. B) Strain-promoted azido-alkyne cycloaddition-mediated conjugation to N_3-F127. n=3.

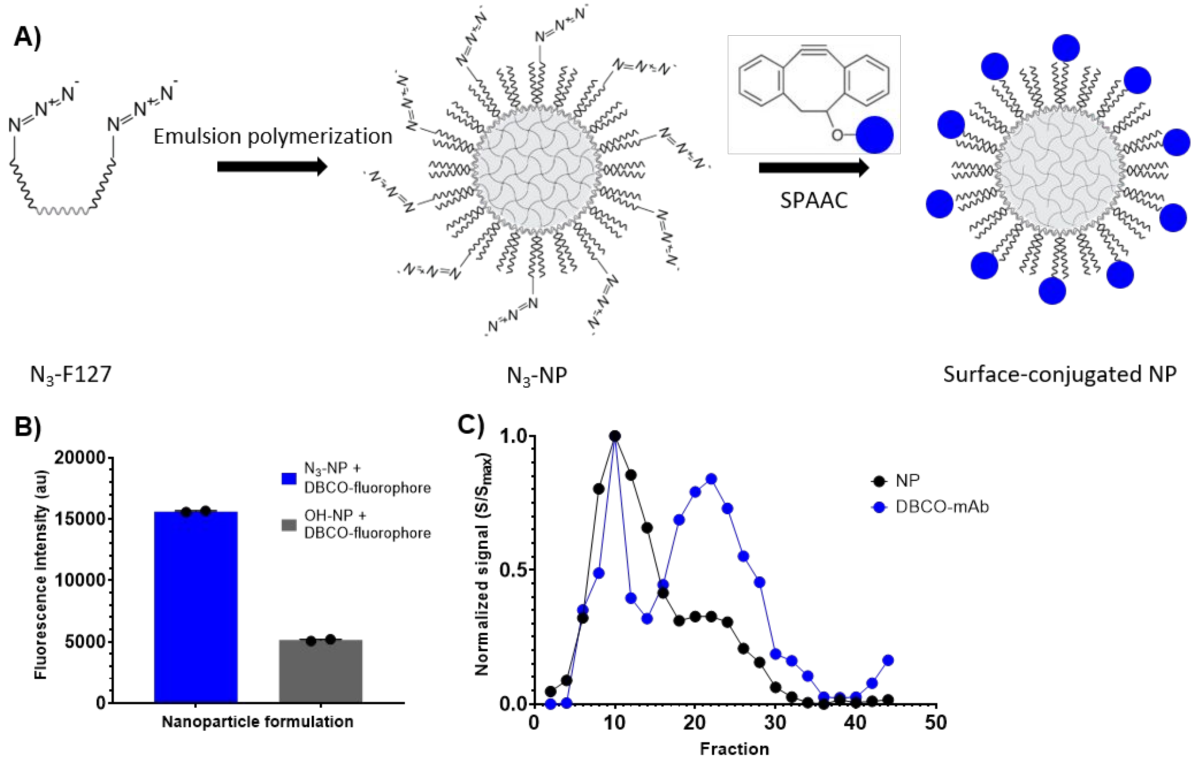

Figure S2. N_3-NP characterization. A) Schematic of N_3-NP synthesis and conjugation. B) Strain-promoted azido-alkyne cycloaddition-mediated conjugation to N_3-NP. n=2. D) SEC elution of N_3-NP and DBCO-functionalized mAb reaction mixture, with the absorbance (NP) and fluorescence (DBCO-mAb) signals normalized by the maximum signal for each elution.

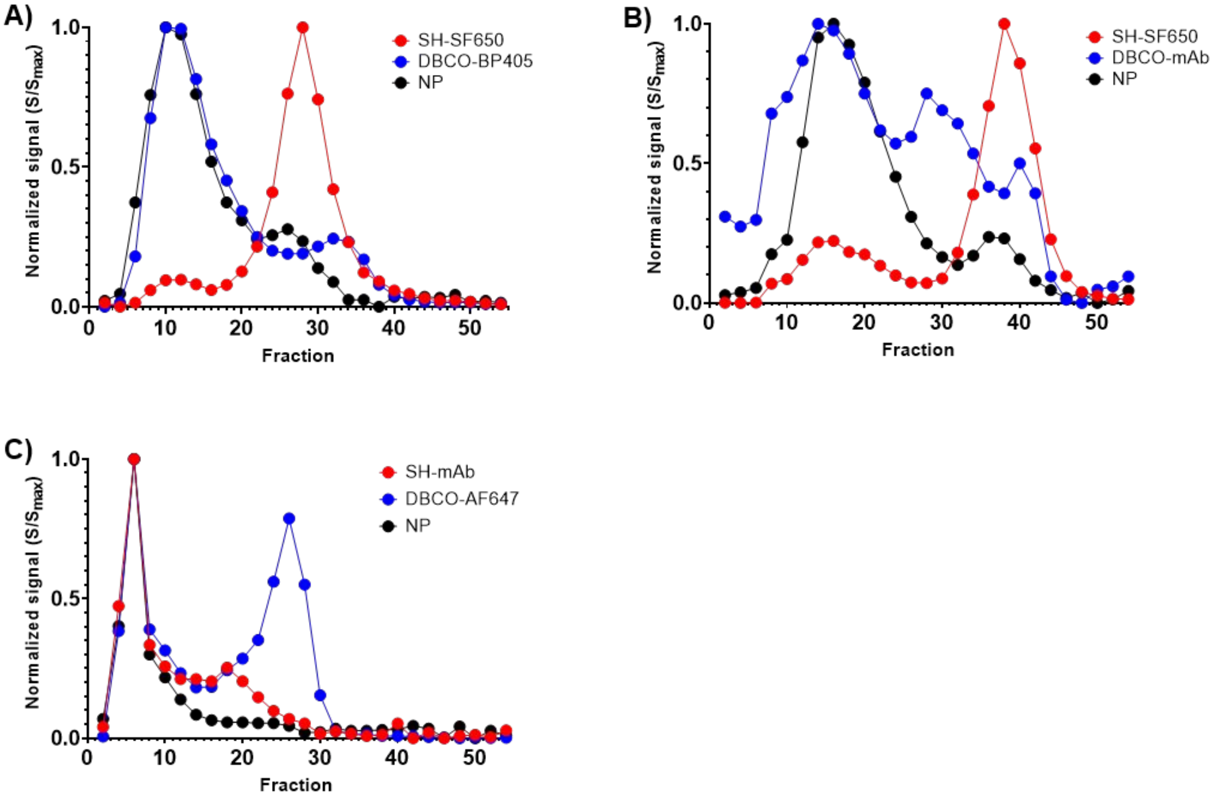

Figure S3. Reaction of homogenous and heterogenous conjugates to dual-functional nanoparticles. A) Representative SEC elution of dual-reactive NP and dual fluorophore reaction mixture. B) Representative SEC elution of dual-reactive NP, thiolated fluorophore, and DBCO-functionalized mAb reaction mixture. C) Representative SEC elution of dual-reactive NP, thiolated mAb, and DBCO-functionalized fluorophore reaction mixture. The absorbance (NP) and fluorescence (SH-SF650, SH-mAb, DBCO-AF647, and DBCO-mAb) signals were normalized by the maximum signal for each elution.

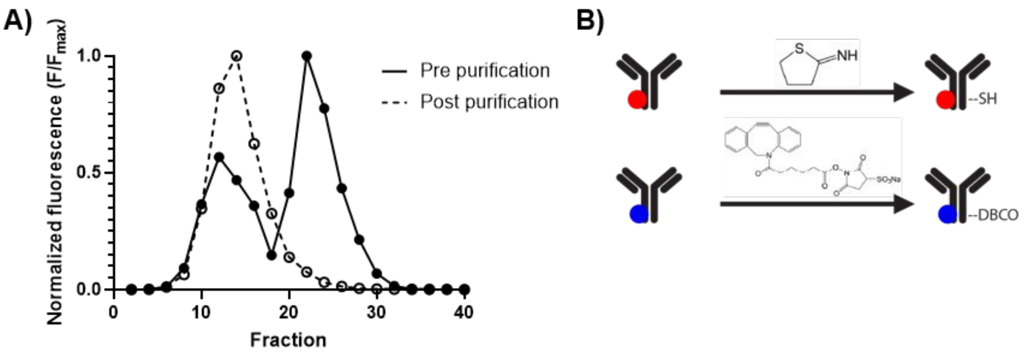

Figure S4. Antibody fluorophore labeling and functionalization. A) Representative SEC elution of fluorophore-labeled mAb before and after purification, with the fluorescence normalized by the maximum fluorescence for each elution. B) Schematic of fluorophore-labeled mAb functionalization with 2-iminothiolane or a DBCO-NHS ester linker.

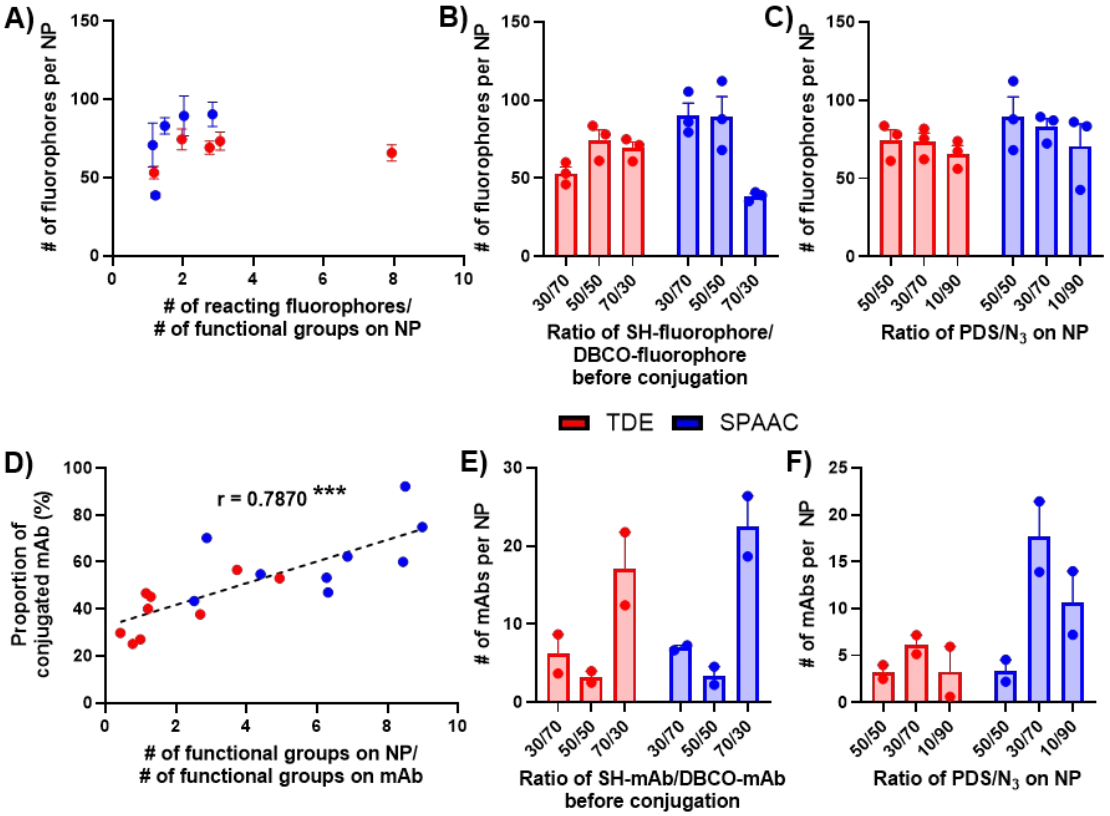

Figure S5. Conjugation characterization for dual-conjugated nanoparticles. A) Number of conjugated fluorophores per NP dependence on the extent of excess reacting fluorophores per NP functional groups. n=3. B-C) Number of conjugated fluorophores per NP for B) 50/50 PDS/N_3-NP conjugating different ratios of functionalized fluorophores and C) NPs with different ratios of functional groups conjugating a 50/50 mixture of functionalized fluorophores. n=3. D) Ratio of conjugated mAb dependence on the extent of excess NP functional groups per reacting mAb functional groups. The dotted line represents linear regression with Pearson coefficient (r), and *** ($p < 0.001$) indicates significant correlation. E-F) Number of conjugated mAbs for E) 50/50 PDS/N_3-NP conjugating different ratios of functionalized mAbs and F) NPs with different ratios of functional groups conjugating a 50/50 mixture of functionalized mAbs. n=2.

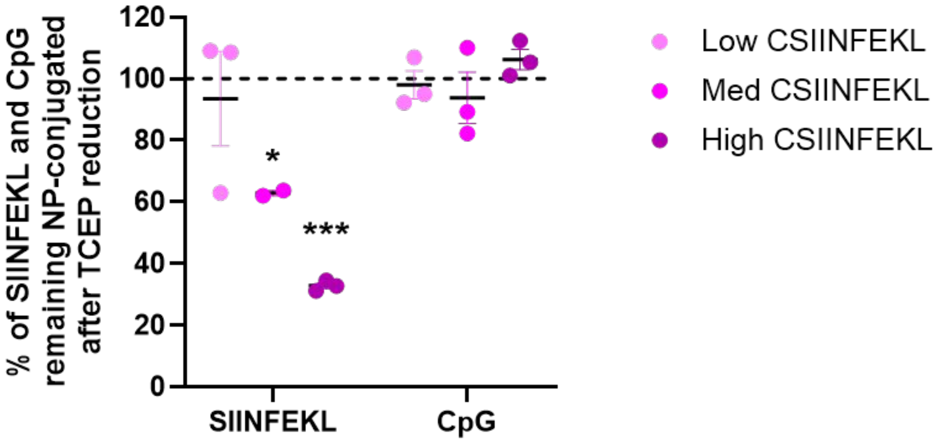

Figure S6. Dual CSIINFEKL/CpG-NP responsiveness to TCEP reduction. For dual CSIINFEKL/CpG-NP, the % of SIINFEKL and CpG remaining conjugated to the NP after TCEP-mediated disulfide reduction. The dashed line represents 100% of the original SIINFEKL or CpG

dose conjugated. * ($p < 0.05$) and *** ($p < 0.001$) indicate significant difference from 100% by one-sample t-test. n=2-3.

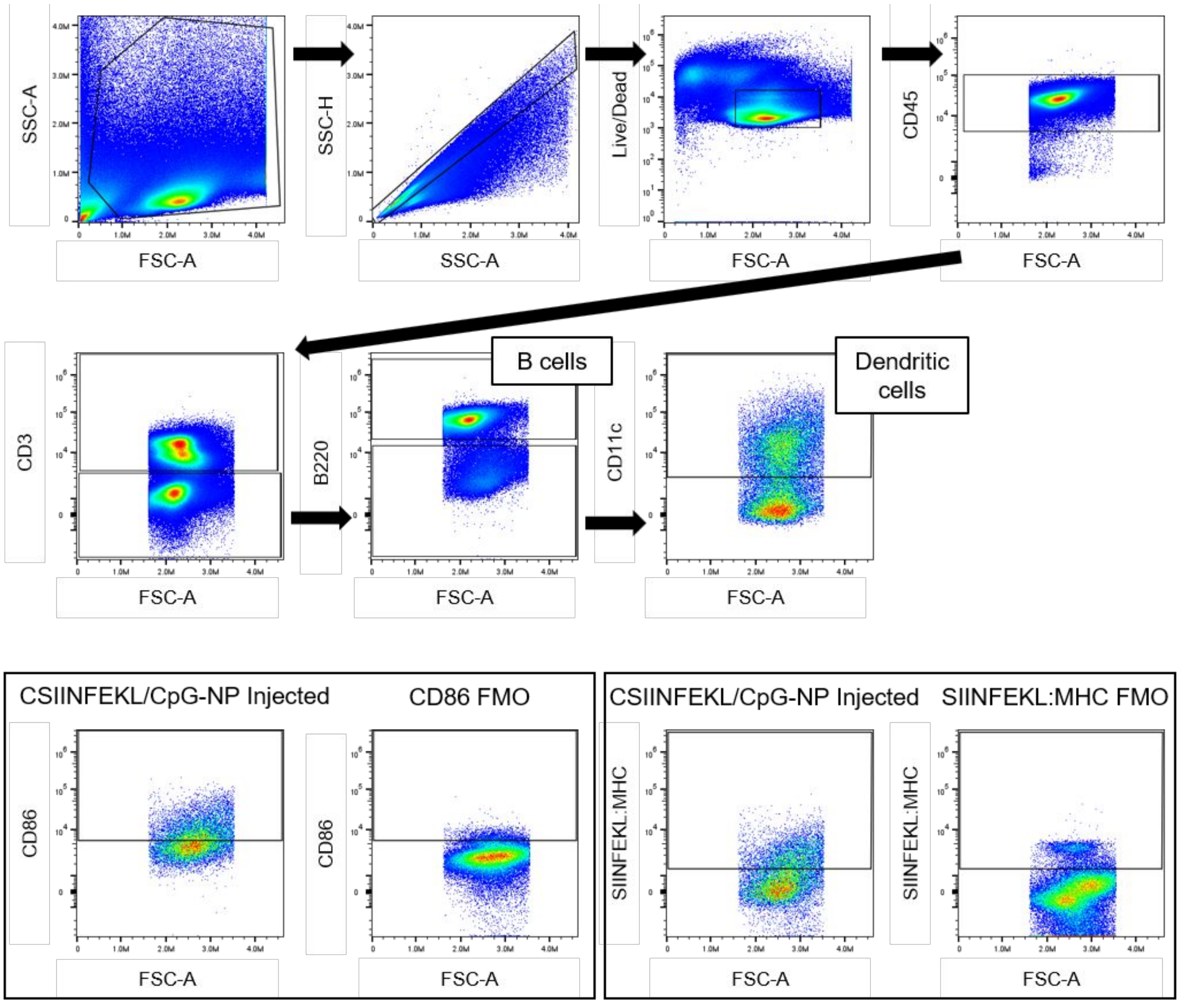

Figure S7. Lymphocyte gating strategy for co-stimulation and antigen presentation.

Table S1: Antibodies used for flow cytometry.

Color	Marker	Clone	Biolegend Cat. #	Dilution
BV510	B220	RA3-6B2	103248	1.25:100
Pe/Cy7	CD11c	N418	117318	0.3:100
Pacific Blue	CD3	17A2	100214	0.25:100
PerCP	CD45	30-F11	103130	0.3125:100
BV650	CD86	GL-1	105035	0.3125:100
PE	H-2K^b:SIINFEKL	25-D1.16	141604	1.25:100
Zombie UV	Viability	N/A	423108	0.125:100
